# First Nationwide Attitude Survey of Japanese Physicians on the Use of Traditional Japanese Medicine (Kampo) in Cancer Treatment

**DOI:** 10.1155/2012/957082

**Published:** 2012-11-22

**Authors:** A. Ito, K. Munakata, Y. Imazu, K. Watanabe

**Affiliations:** ^1^Aoyama Pharmacy, 2-10-9 Minami-Aoyama, Minato-ku, Tokyo 107-0062, Japan; ^2^Center for Kampo Medicine, Keio University School of Medicine, 35 Shinanomachi, Shinjuku-ku, Tokyo 160-8582, Japan; ^3^Pharmaceutical Education Research Center, School of Pharmacy, Kitasato University, 5-9-1 Shirokane, Minato-ku, Tokyo 108-8641, Japan

## Abstract

The aim of this nationwide survey was to investigate the use of Kampo medicine by Japanese physicians who worked in the core cancer treatment hospitals which were designated by Ministry of Health, Labour and Welfare. Among the 900 physicians surveyed, 92.4% reported having prescribed Kampo medications, of whom 73.5% reported having prescribed them for cancer patients. Despite this high percentage and the finding that only 9.7% of the physicians reported that they considered Kampo medications to be harmful, only 23.1% of the physicians expressed high expectations of the efficacy of Kampo medicine in tumor suppression and the exertion of immunostimulatory action. In contrast, many cancer patients have expressed the belief that Kampo medications can suppress tumor growth, and several studies have reported that they exert immunostimulatory action. To resolve this discrepancy in patient and physician expectations and to clarify the research findings, further research into the effectiveness and harmfulness of Kampo medicine in cancer treatment is warranted.

## 1. Introduction

The use of traditional medicine (TM) and complementary and alternative medicine (CAM), also referred to as “non-conventional” medicine, has increased significantly throughout the world over the past several decades. The World Health Organization (WHO) reported that between 70% and 90% of the populations of Canada, France, Germany, and Italy have used some form of TM or CAM and that 110 of the 193 WHO member states reported having some type of policy in place regarding regulation and/or registration of traditional medications in 2007 [1]. In comparison, fewer than 15 countries were able to make the same claim in 1986 [1]. Regarding other parts of the developed world, which has faced an increasing number of deaths due to cancer as a result of a consequence of a confluence of factors, including population growth and population aging, several surveys indicate that more than 40% of US patients in general and more than 60% of US cancer patients use TM or CAM therapies [1, 2].

TM and CAM use is not confined to the developed world. Indeed, between 70% and 95% of citizens in the majority of developing countries, especially those in Asia, Latin America, and the Middle East, have been reported to use TM, including traditional herbal medications, to treat primary health conditions and to promote their health. In Brazil, it was reported that 89% of patients with cancer used TM/CAM products [1]. 

In Asia, the Asia Cancer Forum was established with the aim of overcoming the common challenges of treating cancer shared by humanity as a whole, including those faced by people in developing countries, who often have little access to expensive allopathic medications. One means of overcoming these challenges may be the use of Kampo, a traditional form of Japanese medicine that developed uniquely within Japan after being transferred from China via the Korean peninsula. Unlike in other countries, including the United States and EU nations, where most herbal preparations are regulated as dietary supplements, Kampo medications are regulated in Japan as both prescription and over-the-counter (OTC) medications. Every physician in Japan can prescribe both Kampo and allopathic medications, which may account for the finding that more than 80% of physicians currently prescribe Kampo medications [3].

Cancer, which has been the leading cause of death in Japan since 1981, currently affects 1 in 2 Japanese men and 1 in 2 Japanese women over the lifetime and accounts for 1 in every 3 deaths. The Japanese government recently presented an overview of the Basic Plan to Promote Cancer Control Programs [4]. In light of the Japanese government's focus on cancer control and the increasing use of TM and CAM, this nationwide survey was conducted to understand the use of Kampo medication by Japanese physicians in core cancer treatment hospitals in the treatment of cancer patients and the perceptions regarding the effectiveness of Kampo medicine. The findings of this study may have important implications for the cancer treatment of not only the Japanese population but also that of populations worldwide.

## 2. Materials and Methods

### 2.1. Sample

Before the initiation of this study, the protocol had been examined and approved by the institutional review board of Keio University School of Medicine. The Japanese Ministry of Health, Labour and Welfare designated core cancer treatment hospitals in which advanced cancer treatments are available under the 3rd-term comprehensive 10-year cancer control strategy (2004–2013). The aim of designation of core cancer treatment hospitals is to provide the effective treatment and care of cancer. The government promoted connecting these designated core cancer treatment hospitals to regional clinics. Under this system, hospitals would perform the primary surgical procedures necessary to treat a disease while the treatment and regional clinics would follow the patient after surgery, chemotherapy, or irradiation. These core cancer treatment hospitals were the object in this study to determine the positioning of Kampo medicine in cancer treatment. 

On January 27, 2010, letters requesting participation in the survey were sent to 392 core cancer treatment hospitals throughout Japan. As of January 2010, they are composed by the prefectural-designated and regional-designated cancer care hospitals and Tokyo-designated cancer treatment hospitals. Before the study began, the necessary number of questionnaires for physicians was estimated by the responsible physician in each core cancer treatment hospital and reported to the Center for Kampo Medicine, Keio University School of Medicine. On February 18, 2010, the estimated number of questionnaires was sent to the cooperative hospitals and the responsible person in each hospital distributed to the physicians. The questionnaires were returned to the Center for Kampo Medicine by the physician before November 2, 2010. After being received, the questionnaires were coded with an identification number to ensure confidentiality. 

### 2.2. Questionnaire

As can be observed in the English translation of the original Japanese-language questionnaire in the Appendix, the questionnaire was comprised of 2 parts. The first part collected background data by asking the physicians to respond to 7 closed items (questions from 1 to 7) regarding the type of medical institution for which they worked, their age, specialty of medicine practiced, forms of cancer treatment provided, and prescription experience with Kampo. The second part collected information regarding their expectations of the effectiveness and harmfulness of Kampo and acupuncture and moxibustion (acu/moxa) therapy by asking them to respond to 4 items (questions from 8 to 11) to which they responded using a scale that ranged from 1 (little expectation of effectiveness/harmfulness) to 5 (great expectation of effectiveness/harmfulness) and provided them with the opportunity to provide additional information in an open-ended manner. 

## 3. Results

### 3.1. Questionnaire Response Rate

Of the 392 cancer hospitals throughout Japan to which the questionnaires were sent, 124 (31.6%) granted permission for their distribution to their cancer physicians. Based on the number of eligible physicians estimated by the responsible physician working for each collaborating hospital, 1,816 questionnaires were sent to cooperative hospitals. Of the 909 questionnaires returned to the Center for Kampo Medicine, and 900 (49.6%) were valid and analyzed. 

### 3.2. Physician Background Data

As can be observed in Tables 1 and 2, which summarize the background data regarding the participating physicians, 616 (68.4%) were working in nonuniversity hospitals and 239 (26.6%) in university hospitals. Regarding age, 35.7% were in their 40s, 25.0% in their 30s, 21.2% in their 50s, 8.9% in their 20s, and 3.4% in their 60s. Regarding specialty of medicine practiced, 13.3% practiced general surgery, 11.4% gastroenterology, and 11.0% abdominal surgery, while the remainder practiced a specialty that was practiced by less than 10% of all physicians. The “other” category includes physicians who checked “other” as their specialty and physicians who practiced a specialty that is practiced by less than 1% of all physicians. Of the 164 (18.2%) physicians who responded that they practiced an “other” specialty, 113 specified their specialty and 29 specified that they were residents. Of the 113 who specified their specialty, 26 (2.9%) specified radiation oncology, 20 (2.2%) palliative care, 19 (2.1%) breast surgery, 17 (1.9%) oral surgery, and 14 (1.6%) anesthesiology. As can be observed in Table 2, 94.0% (846) of all the physicians reported that they provided cancer treatment. Regarding the form of treatment, 684 (80.9%) reported that they provided chemotherapy, 458 (54.1%) surgical therapy, 340 (40.2%) palliative care, 171 (20.2%) endoscopic therapy, 166 (19.6%) radiation therapy, and 8 (0.9%) adoptive immunotherapy. Of the 25 physicians who specified “other,” 22 specified the form of therapy, of whom 9 (1.0%) specified interventional radiology, 3 (0.3%) diagnostic imaging, and 3 (0.3%) hematopoietic stem-cell transplantation. 

As for the education level of Kampo, because the Kampo education in medical schools started officially in 2001, most of the participated physicians had not been educated in medical schools when considering the ages of respondents. Also the number of board certified Kampo specialists are only 2450 among 280,000 physicians in Japan. This means that majority of the respondents were nonspecialists. And they are assumed to prescribe Kampo medicines according to the modern diagnosis.

### 3.3. Prescription Experience

**Figure 1:**
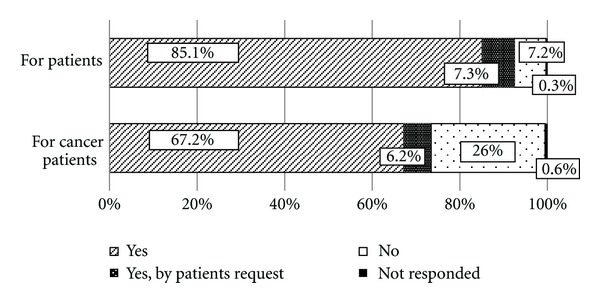
Prescription experience of physicians who provide cancer treatment (*N* = 900).

As shown in Figure 1, 832 physicians (92.4%) reported having prescribed Kampo medications to patients, of whom 66 (7.3%) had done so upon patient request. Among these 832 physicians, 661 (73.4%) had prescribed Kampo medications for cancer treatment, of whom 56 (6.2%) had done so upon patient request. 

### 3.4. Expectation of Effectiveness of Kampo Medicine

**Figure 2:**
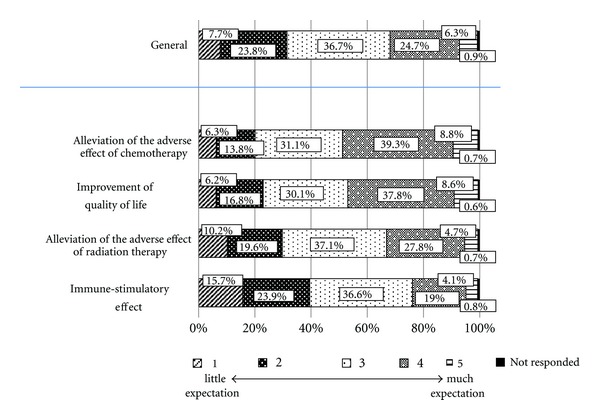
Expectation of effectiveness of Kampo medicine among physicians who provide cancer treatment (*N* = 900).

Regarding their general expectation of the effectiveness of Kampo medicine, 36.7% of the physicians indicated level 3, 24.7% level 4, 23.8% level 2, 7.7% level 1, and 6.3% level 5 (Figure 2). Regarding their expectation of the ability of Kampo medicine to counter the adverse effects of chemotherapy and improve quality of life, 39.3 and 37.8%, respectively, indicated level 4; 31.1 and 30.1%, respectively, level 3; 13.8 and 16.8%, respectively, level 2. Regarding their expectation of the ability of Kampo medicine to counter the adverse effects of radiation, 37.1% indicated level 3, 27.8% level 4, and 19.6% level 2. Regarding their expectation of Kampo medicine to yield an immune-stimulatory effect, 36.6% indicated level 3, 23.9% level 2, and 19.0% level 4. 

Additionally 14 physicians described their comments of expectations for the effectiveness of Kampo medication, for example, the improvement of digestive discomfort, peripheral neuropathy, hiccough, insomnia, or mental stability. 

### 3.5. Perceptions of Harmfulness of Kampo Medicine

**Figure 3:**
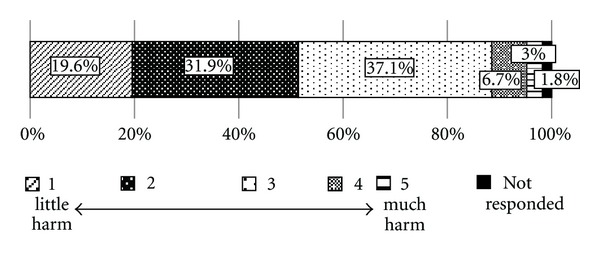
Expectation of harmfulness of Kampo medicine among physicians who provide cancer treatment (*N* = 900).

Regarding perception of the harmfulness of Kampo medicine, 37.0% the physicians indicated level 3, 31.9% level 2, 19.6% level 1, 6.7% level 4, and 3.0% level 5 (Figure 3). Of the 71 physicians who provided additional information regarding their perception and observation of the harmfulness of Kampo medicine, 33 specified that patients experience difficulty taking Kampo medications because of their bad taste and smell and the high volume of medication contained in each dosage; 9 described observation of lung dysfunction, including interstitial pneumonitis and liver dysfunction, with its administration; 4 described observation of pseudohyperaldosteronism (hypokalemia); 3 described observation of drug eruptions, gastritis, allergic reactions, and diarrhea; 3 described observation of adverse effects that are also observed with the administration of allopathic medications. Two physicians also expressed 2 very interesting opinions: (1) because patients overestimate the effects of Kampo medications, they take them too readily and physicians prescribe them too readily and (2) the adverse effects of Kampo medicine cannot be determined because it is unclear whether the adverse effects reported are truly the results of Kampo medicine or rather the results of conventional cancer treatment.

### 3.6. Perception of Effectiveness of Acupuncture and Moxibustion Therapy

**Figure 4:**
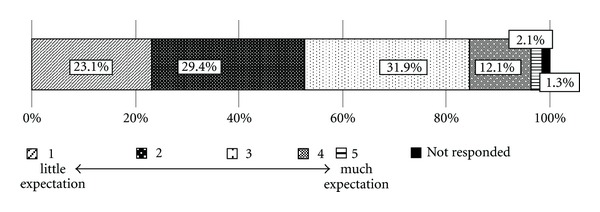
Expectation of effectiveness of acupuncture and moxibustion therapy among physicians who provide cancer treatment (*N* = 900).

Regarding their perception of acu/moxa therapy, 31.8% the physicians indicated level 3, 29.4% level 2, 23.1% level 1, 12.1% level 4, and 2.1% level 5 (Figure 4). Among the 40 physicians who provided additional information regarding their perceptions, 7 described observation of an analgesic effect, 4 described observation of a relaxing effect, 7 reported that they required evidence of the clinical effectiveness before prescribing acu/moxa therapy, and 14 reported that they were not sufficiently familiar with acu/moxa therapy to comment upon it. 

## 4. Discussion

Kampo is a traditional Japanese medicine that shares origins and characteristics with traditional Chinese and Korean medicine. However, whereas medical practitioners in China and Korea practice traditional and allopathic medicine separately, as indicated by the need to obtain a separate license to practice each form, all physicians in Japan who have passed the unified national exam for the practice of allopathic medicine can prescribe both traditional and allopathic medications. A recent report indicates that Japanese physicians indeed do so, having found that 80% of Japanese physicians prescribe Kampo medications [3], a percentage in accordance with the percentage of physicians (92.4%) in this study who reported having prescribed Kampo medications. In addition to prescribing Kampo medications, physicians may recommend that their patients receive acu/moxa therapy from one of the approximately 85,000 practitioners who have passed a national exam qualifying them to provide it.

According to UN and WHO data, Japan has the longest life expectancy of any country in the world and the highest healthy life expectancy of the 192 WHO member states [5, 6]. These positive outcomes may reflect the fact that, in accordance with Japanese law, all medical services in Japan are provided by a national health insurance program at standardized prices to all patients [7]. Cancer, the second leading cause of death globally [8], is an especially pressing concern in Japan, having been the leading cause of death since 1981 and currently accounting for 30% of all deaths [9]. As all physicians in Japan can prescribe both conventional and traditional medications, they can treat cancer patients simultaneously with allopathic and traditional therapies. The results of this study also supported that 73.4% of the physicians who participated in this survey had prescribed Kampo medications for cancer patients.

 Interestingly, some physicians had prescribed Kampo medicines upon patients' request. This means that the Kampo treatment is well accepted by patients too. Takeda et al. reported that 22.9% of gynecological cancer patients had used Kampo medicines [10], and they demanded Kampo treatment because they believed that “Kampo offers relief of symptoms,” “fewer side effects than Western-style medicine,” and “is not less effective than Western-style medicine” than nonusers. 

Although only 31.0% of the physicians reported that they had high expectations that Kampo therapy would effectively treat cancer patients in terms of tumor suppression and promotion of immunostimulatory action, many expressed high expectations that Kampo therapy could alleviate the adverse effects of chemotherapy and improve quality of life. 

This expectation has been supported by the findings of several studies: hangeshashinto prevented diarrhea with the administration of irinotecan (CPT-11) [11]; rikkunshito improved anorexia with the administration of cisplatin (CDDP) [12]; goshajinkigan improved peripheral neuropathy with the administration of oxaliplatin (L-OHP) [13] or FOLFOX [14]. In one study, Hyodo et al. found that of the 44.6% of Japanese cancer patients who reported using CAM, among whom 69.0% reported using mushrooms (*Agaricus*, 60.6% and active hexose correlated compound [AHCC], 8.4%) and 7.1% Kampo medications, 67.1% expressed expectation of suppression of tumor growth with these forms of treatment [15]. However, the Kampo medications they examined in their study were primarily OTC rather than prescription medications. 

While several studies have reported that providing concomitant Kampo and conventional cancer therapy has a significant positive effect on survival time [16–18], a smaller number of physicians in this study reported expectation of an immunostimulating effect with Kampo therapy compared to the number of physicians who reported expectation of other effects. 

Several physicians asserted that more clinical evidence of the effectiveness of Kampo must be accumulated to develop further understanding of Kampo medicine. Watanabe et al. suggested that individualized and long-term Kampo clinical trial design is more suitable to show the benefit of Kampo treatment [19]. 

With regard to expectation of the harmfulness of Kampo medicine, fewer than 10% of the physicians reported expectation of much harm (level 4 or 5), with most indicating that they considered Kampo medicine to pose little risk. Several indicated that the primary disadvantages of Kampo medicine are its bad taste and smell and the high volume of medication that must be taken at each dosage [20]. Whereas traditional Kampo medicine generally takes the form of decoctions of herbs, extract preparations in granular form, the form that most physicians prescribe, are now available. One study found that, among the 98% of patients at one university hospital who reported taking Kampo medications in granular form, approximately 30% experienced difficulty in taking them due to reasons such as their bad taste and therefore preferred taking Kampo medications in tablet over granular form [20]. Unfortunately, only 6% of Kampo medications are currently available in capsule or tablet form. 

Several physicians expressed concern that Kampo medicine may cause liver dysfunction or interstitial pneumonitis. However, liver dysfunction can also be induced by many allopathic medications, which generally do so at a higher rate than Kampo medications. The MHLW found that whereas 4.7% of all cases of drug-induced liver dysfunction in Japan can be attributed to Kampo medications, 22% can be attributed to antibiotics [21]. While the pathogenesis of drug-induced interstitial pneumonitis is poorly understood, it has been found to be induced by not only Kampo but also many allopathic medications, with the rate of bleomycin-induced interstitial pneumonitis reported to be 8–10%, mitomycin C-induced interstitial pneumonitis to be 2–12%, and methotrexate-induced interstitial pneumonitis to be 7% [22, 23]. There have also been reports of death due to interstitial pneumonitis induced by shosaikoto or gefitinib, the former of which is a Kampo medication with a less than 0.1% rate of interstitial pneumonitis among its users while the latter is an anticancer drug with a reported 5.8% rate [24].

Several physicians expressed the belief that the use of Kampo medications poses the risk of the same adverse events as does the use of allopathic therapy and, due to the lack of reporting of these adverse events, that they are too readily prescribed by physicians and taken by patients, leading to their overuse. In many cases, it is difficult to detect the causal drug in cancer treatment because medications are combined. The accumulation of the adverse reactions and the well-designed clinical studies are necessary to understand the true benefits and risks of Kampo medication in cancer treatment and care.

With regard to acu/moxa therapy, more than half of the physicians indicated that they had little expectation of its effectiveness in cancer treatment. This finding may reflect the fact that although Japanese physicians can prescribe acu/moxa therapy for their cancer patients, most do not do so. This finding also indicates that despite reports that acu/moxa may improve quality of life for cancer patients [25], few physicians prescribe it, likely due to their lack of familiarity with it as a form of treatment. Further study of the effectiveness of acu/moxa therapy in cancer treatment is thus warranted.

The fact that many physicians responded to the questionnaire items by selecting “3” may reflect a weakness in using a 5-point Likert scale. To overcome this weakness, future studies should use questionnaires in which the respondents are requested to explain their reason for selecting “3” as a response to a questionnaire item. Future studies should also examine private clinics as well as public medical institutions. 

## 5. Conclusions

Analysis of the questionnaire responses revealed that many physicians have prescribed Kampo medications to their cancer patients, which may reflect that they have little expectation of their harmfulness. However, it may also indicate that physicians have little expectation of their effectiveness in suppressing cancer growth. Many physicians reported that their primary expectations for Kampo therapy are alleviation of the adverse effects of chemotherapy and improvement of quality of life. In contrast, many cancer patients have reported their belief that Kampo therapy can suppress tumor growth, a belief supported by several studies that have reported that Kampo medications may exert an immunostimulating effect. Further research into the effectiveness of Kampo medicine as a form of cancer therapy is warranted to bridge the divide between the expectations of cancer patients and their physicians.

## Figures and Tables

**Table 1:** Physician background characteristics (*N* = 900).

Characteristic	No.	%
Worksite		
Hospital	616	68.4
University affiliated hospital	239	26.6
No response	45	5.0

Age, years		
20≦	80	8.9
30≦	225	25.0
40≦	321	35.7
50≦	191	21.2
60≦	31	3.4
70≦	0	0.0
Not responded	52	5.8

Medical specialty*		
General surgery	120	13.3
Gastroenterology	103	11.4
Abdominal surgery	99	11.0
Respiratory medicine	74	8.2
Hematology	68	7.6
Obstetrics and gynecology	55	6.1
Urology	42	4.7
Thoracic surgery	38	4.2
Medical oncology	33	3.7
General internal medicine	27	3.0
Otorhinolaryngology	27	3.0
Dermatology	23	2.6
Neurosurgery	18	2.0
Endocrine surgery	18	2.0
Orthopedic surgery	18	2.0
Psychosomatic medicine	16	1.8
Pediatrics	13	1.4
Other^†^	204	22.7

*The selection of more than one specialty was possible.

^†^“Other” includes physicians who checked “other” or a specialty that is practiced by less than 1% of all physicians.

**Table 2:** Experience with treatment of cancer patients (*N* = 900).

	No.	%
Treats cancer patients		
Yes	846	94.0
No	51	5.7
No response	3	0.3

Treated with therapy^∗†^		
Chemotherapy	684	80.9
Surgical therapy	458	54.1
Palliative care	340	40.2
Endoscopic therapy	171	20.2
Radiation therapy	166	19.6
Adoptive immunotherapy	8	0.9
Other	25	3.0
No response	6	0.7

*The selection of more than one specialty was possible.

^†^The rate (%) is based on the number of physicians who responded “yes.”

## Appendix

1. Please specify your work site.
  □ A hospital
  □ A university-affiliated hospital
2. Please specify your age.
  □ 20–29
  □ 30–39
  □ 40–49
  □ 50–59
  □ 60–69
  □ Over 70
3. Please specify which specialties you practice. You may specify more than one.
  □ General internal medicine
  □ Gastroenterology
  □ Endocrinology
  □ Neurology
  □ Nephrology
  □ Collagen disease
  □ Respiratory medicine
  □ Hematology
  □ Oncology
  □ Pediatrics
  □ Psychosomatic medicine
  □ General surgery
  □ Cardiovascular surgery
  □ Neurosurgery
  □ Ophthalmology
  □ Otorhinolaryngology
  □ Endocrine surgery
  □ Thoracic surgery
  □ Abdominal surgery
  □ Vascular surgery
  □ Pediatric surgery
  □ Urology
  □ Dermatology
  □ Plastic surgery
  □ Obstetrics and gynecology
  □ Orthopedic surgery
  □ Other ( )
4. Have you ever treated cancer patients?
  □ Yes
  □ No
5. If you answered yes to question 4, which therapies have you used?
  □ Chemotherapy
  □ Radiation therapy
  □ Surgical therapy
  □ Endoscopic therapy
  □ Palliative care
  □ Adoptive immunotherapy
  □ Other ( )
6. Have you ever prescribed Kampo medications for any condition?
  □ Yes
  □ No
  □ Yes, upon patient request
7. Have you ever prescribed Kampo medications for cancer treatment?
  □ Yes
  □ No
  □ Yes, upon patient request
8. Please specify your expectation of the effectiveness of Kampo medicine for cancer treatment.
10. Please specify your expectation of the effectiveness of Kampo medicine for the following aspects of cancer treatment.
  ■ Immunostimulatory effect
11. ■ Alleviation of the adverse effects of chemotherapy
12. ■ Alleviation of the adverse effects of radiation therapy
13. ■ Improvement of quality of life
14. Please express your opinions regarding the effectiveness of Kampo medicine in cancer treatment.
15. Please specify your expectation of the harmfulness of Kampo medicine in cancer treatment.

Please express your opinions regarding the harmfulness of Kampo medicine in cancer treatment.
(11) Please specify your expectation of the effectiveness of acupuncture and moxibustion in cancer treatment.
Please express your opinions regarding the effectiveness of acupuncture and moxibustion in cancer treatment.
